# Cognition and Brain System Segregation in Pediatric Brain Tumor Patients Treated with Proton Therapy

**DOI:** 10.14338/IJPT-22-00039.1

**Published:** 2023-07-24

**Authors:** Anna V. Dowling, Benjamin A. Seitzman, Timothy J. Mitchell, Michael Olufawo, Donna L. Dierker, Hari Anandarajah, Ally Dworetsky, Alana McMichael, Catherine Jiang, Dennis L. Barbour, Bradley L. Schlaggar, David D. Limbrick, Jennifer M. Strahle, Joshua B. Rubin, Joshua S. Shimony, Stephanie M. Perkins

**Affiliations:** 1 Department of Radiation Oncology, Washington University School of Medicine, St. Louis, MO, USA; 2 Mallinckrodt Institute of Radiology, Washington University School of Medicine, St. Louis, MO, USA; 3 Department of Pediatrics, Washington University School of Medicine, St. Louis, MO, USA; 4 Department of Neurology, Washington University School of Medicine, St. Louis, MO, USA; 5 Department of Biomedical Engineering, Washington University School of Medicine, St. Louis, MO, USA; 6 Kennedy Krieger Institute, Baltimore, MD, USA; 7 Department of Neurological Surgery, Washington University School of Medicine, St. Louis, MO, USA; 8 Department of Neuroscience, Washington University School of Medicine, St. Louis, MO, USA

**Keywords:** proton therapy, neurocognitive, resting state, brain tumor, late effects

## Abstract

**Purpose:**

Pediatric brain tumor patients often experience significant cognitive sequelae. Resting-state functional MRI (rsfMRI) provides a measure of brain network organization, and we hypothesize that pediatric brain tumor patients treated with proton therapy will demonstrate abnormal brain network architecture related to cognitive outcome and radiation dosimetry.

**Participants and Methods:**

Pediatric brain tumor patients treated with proton therapy were enrolled on a prospective study of cognitive assessment using the NIH Toolbox Cognitive Domain. rsfMRI was obtained in participants able to complete unsedated MRI. Brain system segregation (BSS), a measure of brain network architecture, was calculated for the whole brain, the high-level cognition association systems, and the sensory-motor systems.

**Results:**

Twenty-six participants were enrolled in the study for cognitive assessment, and 18 completed rsfMRI. There were baseline cognitive deficits in attention and inhibition and processing speed prior to radiation with worsening performance over time in multiple domains. Average BSS across the whole brain was significantly decreased in participants compared with healthy controls (1.089 and 1.101, respectively; *P* = 0.001). Average segregation of association systems was significantly lower in participants than in controls (*P* < 0.001) while there was no difference in the sensory motor networks (*P* = 0.70). Right hippocampus dose was associated with worse attention and inhibition (*P* < 0.05) and decreased segregation in the dorsal attention network (*P* < 0.05).

**Conclusion:**

Higher mean dose to the right hippocampus correlated with worse dorsal attention network segregation and worse attention and inhibition cognitive performance. Patients demonstrated alterations in brain network organization of association systems measured with rsfMRI; however, somatosensory system segregation was no different from healthy children. Further work with preradiation rsfMRI is needed to assess the effects of surgery and presence of a tumor on brain network architecture.

## Introduction

With modern therapy, most patients with pediatric brain tumors survive their diagnosis. However, cure often results in significant cognitive, behavioral, and/or neurologic deficits that are difficult to predict and treat. Decreases in measures of overall intelligence have long been documented in survivors, and more recent studies have demonstrated deficits among this population in core cognitive domains such as attention, working memory, and processing speed [1–4]. In adulthood, these patients are less likely to marry or to pursue secondary education and are more likely to be unemployed than noncancer controls or siblings [5–7]. Despite treatment advances, there has been little progress in understanding the mechanism of cognitive injury or the development of efficacious restorative therapies. Additionally, although young age, hydrocephalus, whole brain radiation, and posterior fossa syndrome are associated with worse cognition, robust predictors and quantitative measures of cognitive outcome are lacking [8, 9].

Advanced functional magnetic resonance imaging (MRI) techniques are currently an untapped resource in understanding the cognitive sequelae in patients with pediatric brain tumor. Resting-state functional MRI (rsfMRI) allows for the study of the spontaneous fluctuations of the human brain at rest, which has revealed a coordinated system of interacting brain regions that correspond to distinct functional networks. These fluctuations in the blood oxygen level–dependent (BOLD) signal are correlated with neuronal activity and are an intrinsic property of the healthy human brain [10, 11]. When fully matured, the brain is organized into a set of segregated and integrated networks that map onto brain activity during a variety of tasks [11]. rsfMRI is able to predict the age of individual healthy children as they follow a normal developmental trajectory [12]. The most prominent and metabolically active network, the default mode network (DMN), is most active when the brain is “at rest” and not engaged in any particular task [13]. Located in posterior cingulate or precuneus cortex, the DMN is involved with mind-wandering, social cognition, perspective shifting, and mental time travel [13]. During task activation, there is significant decreased activity in the DMN with increased activation of networks such as the frontoparietal and dorsal attention networks. Evidence supports that this network segregation of opponent networks begins in childhood with increased segregation associated with higher cognitive scores and lower risk of attentional deficit [14–16]. Just as the segregation and integration of networks is important for development, a loss of this segregation is associated with increasing age [17, 18]. In a work by Chan et al [17], older adults had decreasing connectivity within networks and increasing connectivity between networks, a defining pattern of decreased brain system segregation (BSS).

rsfMRI offers a noninvasive evaluation of brain activity and connectivity and provides a task-free assessment of the brain network organization that subserves human cognition. We hypothesize that advanced functional imaging provides novel information regarding the changes in brain architecture and the relationship of brain organization with cognitive performance. We prospectively obtained baseline and follow-up cognitive testing and rsfMRI in a cohort of patients with pediatric brain tumor treated with proton beam radiation therapy. The NIH (National Institutes of Health) Toolbox Cognitive Domain (NIH TCD) was used for cognitive testing because it provides a flexible and easy-to-use platform for cognitive assessment in children while providing age-adjusted scores and normative means [19, 20]. The tests provide performance results in the following domains: attention and inhibition, episodic memory, working memory, receptive language, oral reading, cognitive flexibility, and processing speed. We hypothesize that (1) acquiring high-quality rsfMRI data during routine clinical imaging is feasible and (2) patients with pediatric brain tumors treated with proton therapy will have abnormal brain network architecture as measured by BSS with relationships between cognitive outcome and radiation dosimetry.

## Materials and Methods

Patients with pediatric brain tumors who were receiving proton therapy at Washington University School of Medicine (participants) were enrolled in a prospective study after informed consent was obtained. All participants underwent cognitive testing using the NIH TCD. Testing was administered in person by a study team member using an iPad device. Testing completion took 30 to 45 minutes. An attempt at baseline testing was made for all participants except those younger than 4 years at diagnosis. Baseline testing was obtained before initiation of radiation or during the first week of treatment. All participants completed follow-up cognitive testing. Patients were offered enrollment to undergo rsfMRI as part of routine clinical imaging. Eligibility criteria included age 4 to 18 years and diagnosis of any primary brain tumor treated with proton therapy. Exclusion criteria included a life expectancy less than 1 year, contraindication to MRI, presence of programmable shunt or dental braces, and inability to complete cognitive testing owing to noncooperation or severe motor or visual deficits. The study was approved by the Washington University Institutional Review Board.

Patient characteristics and clinical factors were gathered from medical records at the time of enrollment. Characteristics collected included participant sex, hydrocephalus at diagnosis, census tract level poverty data, age at radiation, and tumor type. Radiation dosimetric data were collected for all participants and included mean brain dose, volume of brain receiving 20 Gy (V20), volume of brain receiving 30 Gy (V30), mean right and left hippocampus dose, and mean right and left temporal lobe dose. Hippocampus and temporal lobe volumes were manually contoured by the attending radiation oncologist.

### Cognitive Testing

Participants performed tests in the default order programmed in the testing software: Picture Vocabulary, Flanker Inhibitory Control and Attention, List Sorting Working Memory, Dimensional Change Card Sort, Pattern Comparison, Picture Sequence Memory, and Oral Reading Recognition. Scores for individual tests were age adjusted and used to calculate 4 composite scores: Early Childhood Composite (Picture Vocabulary, Flanker, Card Sort, and Picture Sequence), Fluid Cognition (Flanker, List Sort, Card Sort, Pattern Comparison, and Picture Sequence), Crystallized Cognition (Picture Vocabulary and Oral Reading Recognition), and Total (all tests).

### Resting-State Functional MRI Acquisition

Participants underwent unsedated rsfMRI during routing clinical imaging. During rsfMRI scans, participants were instructed to lie still with eyes open during a period of rest. No auditory or visual stimuli were presented. In total, 14 minutes of additional scan time for rsfMRI were added to the clinical scan. A Siemens Trio 3T scanner (Munich, Germany) was used to acquire T1-weighted, T2-weighted, and BOLD contrast sensitive images. BOLD images were acquired via gradient-echo echo-planar imaging with a repetition time of 2.07 seconds, an echo time of 25 ms, and a flip angle of 90°. Two 7-minute runs were obtained, covering the brain in 36 slices (4 mm^3^ isotropic voxels) [11, 21].

Image processing was completed by using the Washington University Neuroimaging Laboratory 4dfp Suite and MATLAB (R2015, MathWorks, Natick, MA). The first 12 frames of each BOLD run were discarded and slice timing correction was applied. All BOLD images for a single participant were aligned to the first frame of the first run, 3D cross-realigned, and normalized. Images were then resampled and registered to the high-resolution T1-weighted and T2-weighted anatomical images and then to an atlas in Talairach space designed for children [22, 23]. Nuisance regression included regressors extracted from individually defined ventricles, white matter, and the global signal, as well as the 6 parameters from motion correction, all of which were subject to Volterra expansion [11, 24, 25]. Segmentation via FreeSurfer 5.3 was used for individual mask definition [26]. Frame censoring was used to further address motion artifacts. Frames exceeding 0.1-mm framewise displacement (after low-pass filtering motion parameters) were censored [26, 27]. Each run was required to have at least 30 low-motion frames, and segments with fewer than 3 continuous frames were also censored. All participants included had 5 or more total minutes of low-motion rsfMRI data.

rsfMRI scans from typically developing, healthy children were used as control data. Control participants (controls) all were native English speakers who had no history of neurologic or psychiatric disease or psychotropic medication use. Controls were recruited from the Washington University campus and the surrounding community [12]. Control scans were acquired on a Siemens Trio 3T scanner with similar acquisition parameters to those used by study participants (Repetition time = 2.5 seconds, echo time&thinsp= 27 ms, flip angle of 90°, 4 mm^3^ isotropic voxels). Images from 18 participants and 98 controls were used in the study.

### Analysis

Cognitive scores in participants were first compared with the standardized age-normalized population mean of 100 with an SD of 15. Two-sided *t* tests were used to compare participant sample scores with the population mean on each component of the NIH TCD. Patient characteristics including age at radiation, sex, presence of hydrocephalus, census tract level poverty status, and radiation dosimetric data were correlated (Pearson) with these components.

Preprocessed rsfMRI data from each participant and control were used to generate correlation matrices composed of all gray-matter voxels in the brain. We applied a previously defined volumetric functional network atlas to sort correlation matrices into functional networks for each patient with a pediatric brain tumor and each control [28]. Brain system segregation was calculated by using the methods of Chan et al [17] as previously described. Individual correlation matrices were Fisher z-transformed and used to calculate BSS, which is defined as the difference between mean within-system and mean between-system correlation as a proportion mean within-system correlation:


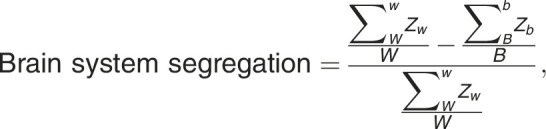
 where *Z* is the Fisher z-transformed correlation value, *w* indicates within-system correlation, and *b* indicates between-system correlation. Brain system segregation was calculated for each individual brain network as well as for all association systems together and all sensory-motor systems together (each including multiple networks). Individual networks consisted of the default mode, visual, frontoparietal, dorsal attention, language, salience, cingulo-opercular, somatomotor dorsal, somatomotor lateral, auditory, anterior temporal, medial temporal, parietal memory, and context memory networks. The association systems included the default mode, frontoparietal, dorsal attention, language, salience, and cingulo-opercular networks. The sensory-motor systems included the visual, auditory, and somatomotor dorsal and lateral systems. The BSS values of participants were compared with those of controls by using standard 2-sided *t* test performed in MATLAB. Benjamini-Hochberg method with a false discovery rate of 0.05 was used to correct for multiple comparisons [29].

## Results

A total of 26 patients were enrolled in the study. Median age at the start of radiation was 9.9 years (range, 2.2-17.9). Baseline cognitive testing was obtained on 20 participants (77%). All patients completed follow-up cognitive testing at a median time of 3.2 years after proton therapy (range, 0.7-7.2). rsfMRI was performed on 18 participants. No participants received sedation during their rsfMRI scan. However, 9 of 26 participants (34%) did receive propofol sedation during their course of proton beam therapy. Participants underwent rsfMRI at a median time of 2.08 years (range, 0.20-4.85) after proton therapy. Median age at time of rsfMRI was 10.5 years (range, 7.2-17.9). Participant demographics, tumor and treatment characteristics are shown in **Table 1**. rsfMRI data from 98 healthy control children (44.9% female) were included for comparison. Average age of the controls was 12.8 years with a range of 9.7 to 17.7 years.

**Table 1.** Patient, diagnosis, treatment, and follow-up data.

**Patient**	**Sex**	**Race**	**Diagnosis**	**Tumor location**	**Treatment**			**Age, y**			**Time since RT, y**	
					**Surgery**	**Radiation extent**	**Chemo therapy**	**At RT**	**At scan**	**At testing**	**At scan**	**At testing**
1	M	W	Medulloblastoma	Cerebellum	Resection	CSI	Yes	14.39	16.39	15.58	2.01	1.20
2	F	AA	Pilocytic astrocytoma	Brainstem	Resection	Focal	No	14.02	17.91	17.92	3.90	3.91
3	M	W	Glioma	Brainstem	Resection	Focal	No	15.54	18.60	18.60	3.07	3.07
4	F	W	Medulloblastoma	Cerebellum	Resection	CSI	Yes	10.68	13.44	13.95	2.77	3.29
5	M	W	Medulloblastoma	Cerebellum	Resection	CSI	Yes	9.68	14.37	12.09	4.69	2.42
6	M	AA	Medulloblastoma	Cerebellum	Resection	CSI	No	17.93	18.92	18.92	1.00	1.00
7	M	W	Medulloblastoma	Cerebellum	Resection	CSI	Yes	11.05	13.91	14.94	2.87	3.90
8	M	W	Medulloblastoma	Cerebellum	Resection	CSI	Yes	7.22	8.87	10.41	1.65	3.19
9	M	W	Craniopharyngioma	Suprasellar	Resection	Focal	No	9.71	11.00	12.08	1.30	2.37
10	M	AA	Craniopharyngioma	Suprasellar	Resection	Focal	No	10.32	12.48	12.00	2.17	1.68
11	M	W	Craniopharyngioma	Suprasellar	Resection	Focal	No	10.28	10.81	11.23	0.54	0.96
12	F	W	Pineal papillary	Pineal	Resection	Focal	No	7.96	8.86	8.86	0.90	0.90
13	M	W	Oligodendroglioma	Thalamus	Biopsy	Focal	No	15.44	16.42	16.42	0.99	0.99
14	M	W	Ependymoma	Cerebellum	Resection	Focal	No	7.96	9.19	11.54	1.23	3.58
15	M	W	Pineoblastoma	Pineal	Resection	CSI	Yes	11.71	16.55	18.89	4.85	7.18
16	M	AA	Ependymoma	Cerebellum	Resection	Focal	No	10.98	12.73	12.73	1.75	1.75
17	F	W	Anaplastic ependymoma	Occipital	Resection	Focal	No	9.16	9.36	10.44	0.20	1.28
18	F	W	Craniopharyngioma	Suprasellar	Resection	Focal	No	10.16	10.70	10.81	0.56	0.66
19	M	W	Medulloblastoma	Cerebellum	Resection	CSI	Yes	4.97	N/A	8.55	N/A	3.59
20	M	W	Medulloblastoma	Cerebellum	Resection	CSI	Yes	5.76	N/A	9.32	N/A	3.57
21	M	W	PNET	Cerebellum	Resection	CSI	Yes	10.08	N/A	14.27	N/A	4.20
22	F	W	Ependymoma	Cerebellum	Resection	Focal	No	2.55	N/A	7.28	N/A	4.73
23	M	W	Anaplastic ependymoma	Occipital	Resection	Focal	Yes	2.52	N/A	7.15	N/A	4.64
24	F	AA	Anaplastic ependymoma	Inferior frontal gyrus	Resection	Focal	Yes	7.51	N/A	10.82	N/A	3.31
25	M	W	Anaplastic ependymoma	Parietal	Resection	Focal	No	2.18	N/A	6.00	N/A	3.83
26	M	W	Medulloblastoma	Cerebellum	Resection	CSI	Yes	9.24	N/A	14.21	N/A	4.97

**Abbreviations:** RT, radiation therapy; W, white; CSI, craniospinal irradiation; AA, African American; N/A, not-applicable; PNET, primitive neuroectodermal tumor.

**Figure 1.**
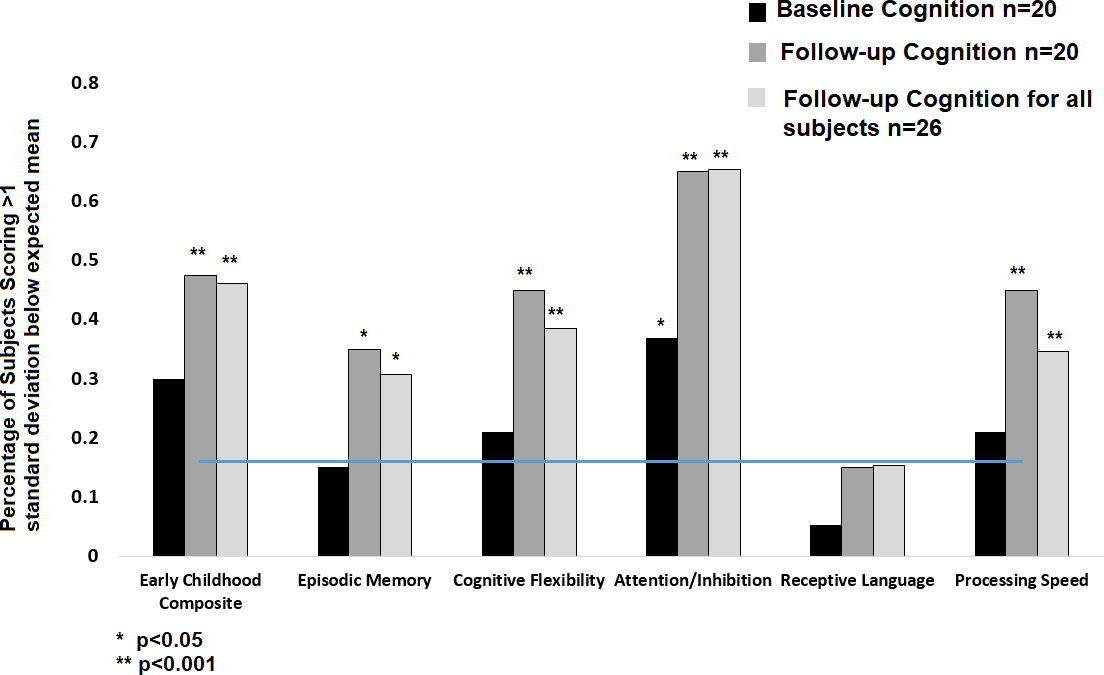
Percentage of participants scoring greater than 1 SD below the mean. Baseline and follow-up cognitive testing are presented. The blue line indicates the expected value of 16% in a normative sample.

Baseline cognitive testing was completed on 20 participants. Six participants did not complete baseline cognitive testing owing to missed testing or inability to complete (n = 3) or patient age was <4 years at baseline (n = 3). At baseline, participants had decreased abilities in attention and inhibition and processing speed with 40% and 47% of participants, respectively, scoring 1 SD below the normative mean (*P* < .05; **Figure 1**). At last follow-up, participants had lower than expected scores in episodic memory (*P* < .02), cognitive flexibility (*P* < .001), attention and inhibition (*P* < .001), and processing speed (*P* < .001). Receptive language remained normal at baseline and follow-up.

For all participants at last follow-up (n = 26), results were compared with the standardized population average score for each test and composite measure by using a 2-tailed *t* test (**Table 2**). Mean participant score was significantly lower than the standardized population mean for early childhood composite, total composite, and fluid composite scores and for the following individual tests: processing speed, cognitive flexibility, and inhibitory control or attention. Participants’ average scores were lowest on the inhibitory control or attention task (mean, 85.5) and on the fluid composite (mean, 84.1). Participants scored highest on average on tests of receptive vocabulary (mean, 98.7), working memory (mean, 98.3), and episodic memory (mean, 96.7).

**Table 2.** NIH Toolbox Cognitive Battery scores at last follow-up: comparisons with age-normalized mean score for healthy children.

**Measure**	**N**	**Mean ± SD**	**% Below 1 SD**	***P*** **value**
Early Childhood Composite	26	86.3 ± 10.8	46.2	<.001
Total Composite score	25	86.5 ± 14.1	52.0	<.001
Crystallized Composite score	25	93.8 ± 12.6	24.0	.021
Fluid Composite score	25	84.1 ± 15.7	48.0	<.001
Oral Reading	25	93.4 ± 13.1	28.0	.019
Episodic Memory	26	96.7 ± 17.4	30.8	.34
Processing Speed	25	86.0 ± 22.9	48.0	<.005
Cognitive Flexibility	26	86.7 ± 11.9	38.5	<.001
Working Memory	25	98.3 ± 13.6	12.0	.54
Inhibitory Control or Attention	26	85.5 ± 14.8	65.4	<.001
Receptive Language	26	98.7 ± 12.7	15.4	.61

**Abbreviation:** NIH, National Institutes of Health.

**Figure 2.**
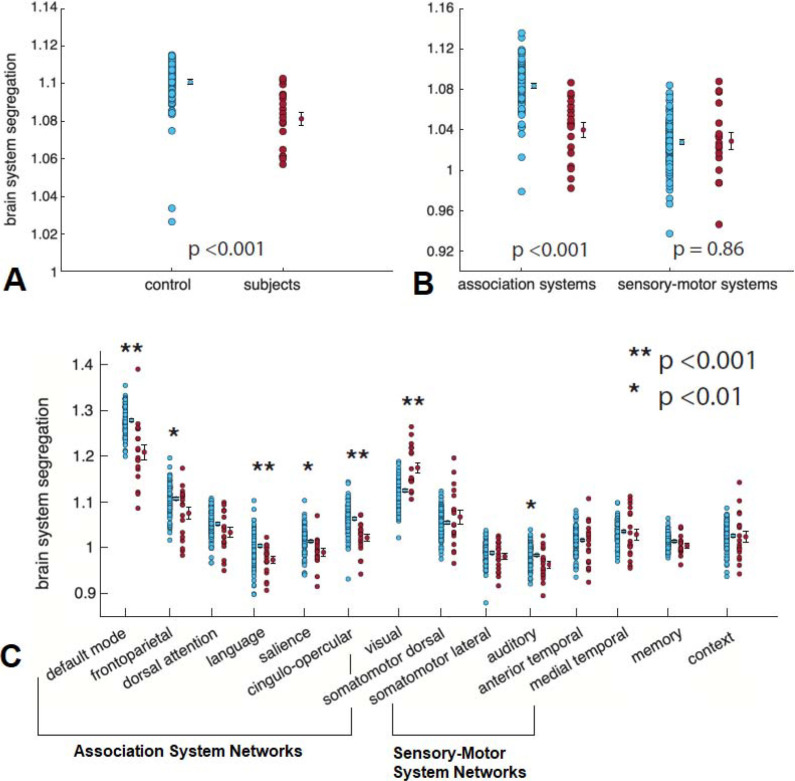
(A) Decreased BSS across the entire brain was seen in participants (n = 18) compared with healthy controls (n = 99). (B) Participants had significant decrease in association system segregation with no difference in sensory-motor system segregation. (C) Decreased individual network segregation in default mode network, language, salience, cingulo-opercular, and auditory networks reach statistical significance. Visual network segregation was statistically increased in participants. Abbreviation: BSS, brain system segregation.

Participants had decreased total BSS as compared with controls with values of 1.082 and 1.101, respectively (*P* = .001; **Figure 2A**). Average segregation of association systems was significantly lower in participants (mean, 1.040) than in controls (*P* < .001). Six of 18 participants (33%) had association system segregation scores >2 SDs below the control mean (*P* < .001). There was no difference in sensory-motor network segregation between the participants and controls (*P* = .86; **Figure 2B**). Brain system segregation was measured in 14 individual brain networks (**Figure 2C**). Participants had decreased segregation in multiple individual networks within the association systems. The most affected network was the default mode network because 9 participants (50%) had default mode network segregation scores >2 SDs below the control mean (*P* < .001). In addition, participants had significantly lower BSS than controls in cingulo-opercular (*P* < .001), language (*P* < .001), salience (*P* < .01), and frontoparietal control (*P* < .05) networks. Participants had significantly higher BSS than controls in the visual system network (*P* < .001) and lower segregation in the auditory network (*P* < .01). All significance levels withstood Benjamini-Hochberg correction with a false discovery rate of 0.05.

**Figure 3.**
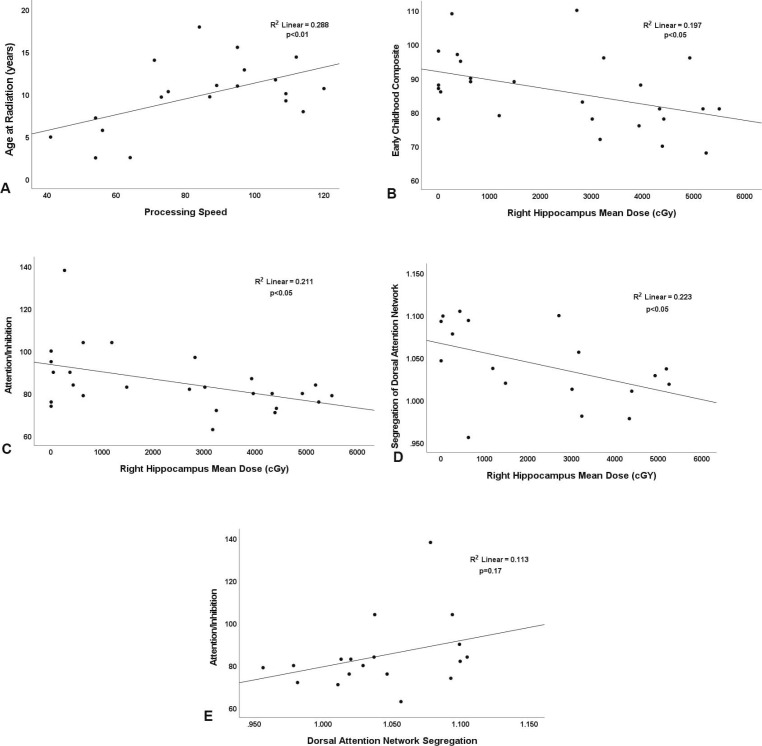
Correlations between age at radiation and processing speed (A), right hippocampus mean dose and Early Childhood Composite score (B), and right hippocampus mean dose and attention and inhibition scores (C) were statistically significant. Increased right hippocampus dose was found to associate with decreased dorsal attention network segregation (D). The relationship between dorsal attention network segregation and attention and inhibition performance did not meet statistical significance (E).

Relationships between patient and clinical factors and cognition and BSS are presented in **Table 3**. Patient sex and presence of hydrocephalus at presentation were tested as binary predictors of Early Childhood Composite score, and tests of processing speed and attention and inhibition. Age at radiation, patient sex, presence of hydrocephalus at diagnosis, socioeconomic status, volume of brain receiving 30 Gy (V30), brain mean dose, left temporal lobe dose, and left hippocampus dose were not associated with worsened cognitive performance or association and default mode network BSS. However, age at radiation therapy was a significant predictor of processing speed, with older age at radiation therapy correlating with higher score (*P* < .01; **Figure 3A**). Attention and inhibition were significantly correlated with both right hippocampus mean dose (*P* = .02; **Figure 3B**) and right temporal mean dose (*P* = .03). There was a trend towards lower attention with higher mean brain dose (*P* = .053). Higher right hippocampus mean radiation dose correlated with lower early childhood composite score (*P* = .02; **Figure 3C**).

**Table 3.** Univariate analysis of patient and clinical factors as predictors of BSS and cognitive measures.

	**Overall BSS**	**BSS of association systems**	**BSS of DMN**	**Early Childhood Composite score**	**Processing Speed score**	**Flanker Attention score**
Sex						
Male	1.080	1.041	1.221	85.4	85.9	85.4
Female	1.086	1.037	1.179	88.6	86.0	85.6
*t*, *P* value	−0.80, .44	0.28, .79	1.12, .28	−0.65, .52	−0.01, .99	−0.02, .98
Hydrocephalus						
Yes	1.079	1.039	1.20	86.2	86.4	84.5
No	1.088	1.043	1.24	86.6	84.5	88.3
*t*, *P* value	–1.20, .25	–0.27, .79	–1.38, .19	–0.08, .93	0.18, .86	–0.57, .58
Age at RT						
*P* value	.33	.61	.72	.28	**.004**	.07
SES						
*P* value	.86	.69	.56	.31	.78	.93
Brain V30						
*P* value	.06	.96	.85	.27	.86	.13
Brain mean dose						
*P* value	.15	.87	.55	.26	.95	.053
Left hippocampus mean dose						
*P* value	.42	.71	.59	.10	.44	.10
Right hippocampus mean dose						
*P* value	.46	.97	.73	**.023**	.86	**.018**
Left temporal mean dose						
*P* value	.11	.78	.83	.30	.79	.09
Right temporal mean dose						
*P* value	.16	.91	.85	.10	.73	**.031**

**Abbreviations:** BSS, brain system segregation; DMN, default mode network; RT, radiation therapy; SES, socioeconomic status as measured by census track poverty level.

Note: Boldface indicates *P* values < .05.

No patient characteristics or dosimetric values were predictive of association BSS or default mode network segregation. However, increased right hippocampus mean dose was associated with decreased dorsal attention network segregation (*P* < .05; **Figure 3D**). The relationship between dorsal attention network segregation and attention and inhibition cognitive score did not reach statistical significance (*P* = .17; **Figure 3E**).

## Discussion

In this cohort of children receiving proton beam radiation for primary brain tumors we observed baseline cognitive deficits in attention and inhibition and processing speed before the start of treatment. With a median follow-up of 3.2 years, participants had worsening attention and inhibition along with lower than expected scores in episodic memory, cognitive flexibility, and processing speed. This was a high-risk cohort for cognitive issues with a high percentage (38%) receiving craniospinal radiation along with the inclusion of children treated who were younger than 5 years (15%). Younger age at radiation was associated with worse processing speed, and higher right hippocampus dose was associated with worse attention and inhibition and a lower early childhood composite score. Compared with healthy children, participants had decreased association BSS as measured by rsfMRI. At the network level, lower BSS was most pronounced in the DMN, frontoparietal, language, salience, and cingulo-opercular networks. BSS of sensory-motor systems as a whole was unchanged when compared with controls. However, the individual visual and auditory networks did demonstrate increased and decreased segregation, respectively, compared with controls. There were no relationships between association system or default mode network segregation with cognitive performance or radiation dosimetry. However, increased right hippocampal dose was associated with decreased dorsal attention network segregation. The relationship between dorsal attention network segregation and attention and inhibition cognitive score did not reach statistical significance.

We have shown the feasibility of obtaining high-quality rsfMRI data in patients with pediatric brain tumors during routine clinical imaging. The resting-state networks are a widely varied system of connected areas of the brain involved in all aspects of brain function including sensory-motor functions and more high-level functioning, such as attention and memory. The assessment of BSS further allows for investigation into interconnected and disconnected (ie, anticorrelated) architecture of the brain. We hypothesized that participants would have abnormal brain network architecture as measured by BSS. We found that association network segregation was significantly decreased in our participants, with association network BSS scores >2 SDs below the control mean for 33% of participants. The default mode network, the largest and most metabolically active network, was the most profoundly affected. However, we did not find relationships between association BSS or default mode segregation and cognitive performance. As the overall cognitive decline observed in patients with pediatric brain tumors can show steady decline for up to a decade [30, 31], it is possible that our follow-up is insufficient and longer follow-up is required. Importantly, childhood is a time in which brain system architecture is established and future work will inform us as to the trajectory of network organization (eg, are participants losing their network architecture or failing to develop normal architecture with age).

Radiation dose to the right hippocampus was predictive of follow-up scores in attention and inhibition and early childhood composite. This finding of the importance of hippocampal dose to cognition is in agreement with others [31, 32]. We also found a significant relationship between right hippocampus dose and segregation of the dorsal attention network, a novel finding merging radiation dosimetric data and advanced functional imaging. The attention and inhibition function, as measured by the Flanker test, was the most affected behavior at last follow-up with 65% of patients scoring greater than 1 SD below the mean of healthy children. The dorsal attention network is involved with the “top-down” processing of attention, and often demonstrates task-positivity and strong anticorrelation with the default mode network (which is most active when not engaged in tasks). This loss of anticorrelation is reflected in the decreased association BSS in our participants, and future work will continue to assess the trajectory with long-term follow-up.

Limitations of our study include the limited number of participants. Additionally, follow-up of 3.2 years is relatively short when assessing overall cognitive outcome for patients with pediatric brain tumors. However, these data do demonstrate appreciable early changes in cognition and brain network organization, yet analysis with longer follow-up is planned. Owing to the COVID pandemic, the study team halted in-person cognitive testing during 2020, thus leading to a delay in cognitive testing in some participants. An additional limitation to our study is that we do not have rsfMRI before radiation to directly assess the difference in preradiation and postradiation BSS. Thus, we are unable to analyze the contribution of proton therapy on BSS versus baseline alterations in BSS due to tumor, hydrocephalus, and/or surgery. The data did not demonstrate associations with mean brain dose, temporal lobe dose, or hydrocephalus and cognitive outcome, although these have been predictive in other publications [32, 33]. This could be due to poor sensitivity of the cognitive measure and/or our limited follow-up time from diagnosis to testing. Our future work will also include children treated with surgery and/or chemotherapy alone to further assess the effect of radiation on resting-state networks. Resting-state networks are represented anatomically in the brain, allowing for import or fusion of the network as an avoidance structure set into clinical planning software for planning optimization. Therefore, as our data set grows, we will evaluate the radiation dose received by individual networks to explore the potential for incorporating rsfMRI networks into the radiation planning process by identifying resting-state networks as avoidance structures during plan optimization.

To our knowledge, these are the first data on rsfMRI in patients with pediatric brain tumors, including the novel assessment of brain network segregation. With a median follow-up of 3.2 years, patients with pediatric brain tumors had notable changes in brain network architecture as measured by rsfMRI. Participants had alterations in association networks, which are thought to reflect high-level functioning, while the sensory-motor networks showed no significant differences when compared with healthy children. Higher radiation doses to the right hippocampus were associated with worse cognitive performance and significant alterations in the dorsal attention network.
